# Insights into the Mechanism of Action of Antipsychotic Drugs Derived from Animal Models: Standard of Care versus Novel Targets

**DOI:** 10.3390/ijms241512374

**Published:** 2023-08-03

**Authors:** Anthony A. Grace, Daniela L. Uliana

**Affiliations:** Departments of Neuroscience, Psychiatry and Psychology, University of Pittsburgh, Pittsburgh, PA 15260, USA; uliana@pitt.edu

**Keywords:** dopamine, antipsychotic, schizophrenia

## Abstract

Therapeutic intervention for schizophrenia relies on blockade of dopamine D2 receptors in the associative striatum; however, there is little evidence for baseline overdrive of the dopamine system. Instead, the dopamine system is in a hyper-responsive state due to excessive drive by the hippocampus. This causes more dopamine neurons to be in a spontaneously active, hyper-responsive state. Antipsychotic drugs alleviate this by causing depolarization block, or excessive depolarization-induced dopamine neuron inactivation. Indeed, both first- and second-generation antipsychotic drugs cause depolarization block in the ventral tegmentum to relieve positive symptoms, whereas first-generation drugs also cause depolarization in the nigrostriatal dopamine system to lead to extrapyramidal side effects. However, by blocking dopamine receptors, these drugs are activating multiple synapses downstream from the proposed site of pathology: the loss of inhibitory influence over the hippocampus. An overactive hippocampus not only drives the dopamine-dependent positive symptoms, but via its projections to the amygdala and the neocortex can also drive negative and cognitive symptoms, respectively. On this basis, a novel class of drugs that can reverse schizophrenia at the site of pathology, i.e., the hippocampal overdrive, could be effective in alleviating all three classes of symptoms of schizophrenia while also being better tolerated.

## 1. Introduction

Schizophrenia is a devastating developmental disorder that arises from an interaction of genetic susceptibility and environmental factors [1,2,3,4], with an incidence of approximately 1–1.5% worldwide [5,6,7]. The illness has a devastating impact on individuals and their families/caregivers [8], striking during late adolescence/early adulthood [5,9]. As a result, there has been substantial effort dedicated to providing a better understanding of the pathophysiology of this disorder as a means to discover more effective treatments. The first treatment developed was based on dopamine antagonism. This was a serendipitous discovery that was made on the basis of an opportune observation that a modified antihistamine, chlorpromazine, was an effective stabilizer for extended surgical interventions [10,11]. In coining the term “neuroleptic” to describe the neural stabilizing action of the drug, Laborit [12] attracted the attention of clinicians working in a mental asylum as a potential pharmacological intervention for a number of at that time untreatable mental disorders. They found that the drug was highly effective for those experiencing schizophrenia [13,14,15,16,17]. It was another 20 years before the drug was proposed to exert its therapeutic action via blockade of dopamine (DA) receptors [18,19], and another 12 years before the DA type 2 receptor (D2) was identified as the binding site of antipsychotic drugs [20]. This, along with evidence of DA releasing drugs exacerbating schizophrenia psychosis [21,22,23], has given rise to the DA hypothesis of schizophrenia [24,25], which has dominated drug development in the treatment of schizophrenia for decades.

Because DA is involved in the psychotic symptoms of schizophrenia as well as in regulation of movement, the combined antipsychotic efficacy and associated neurological side effects were considered to be necessary for the actions of these drugs, which was termed the neuroleptic profile [11,26]. However, with the discovery of clozapine and other second-generation antipsychotics, it was discovered that one can separate pharmacologically the antipsychotic properties from the neurological, or “extrapyramidal,” side effects [27,28,29]. Interestingly, this was not due to the development of more selective pharmacological agents, but instead relied on actions off-target from the D2 receptor, most prominently the serotonergic 5HT2 receptor [30] and possibly anticholinergic properties [31]. Thus, although these second-generation compounds targeted multiple receptors with various affinities, the drugs still needed to be administered at a dose that caused 60–80% occupancy of the D2 receptor [32,33,34,35].

How did these drugs achieve their action? This was a quandary, since despite the known efficacy at the D2 receptor, there was a little evidence for a dysfunction within the DA system itself; particularly when compared to the psychotomimetic effects of amphetamines that produce psychosis by increasing DA overflow 20–30-fold over baseline [36,37], which was clearly not present in the schizophrenia patient [21]. Furthermore, the antipsychotic drugs did not have a pharmacological profile consistent with acute actions, in that the drugs typically had to be administered several times to achieve therapeutic actions [38], which was unusual since one would predict greatest efficacy at the first dose with the development of tachyphylaxis with additional doses. Instead, these drugs showed increased efficacy without the need to dramatically increase drug doses to adjust for homeostatic compensation to D2 blockade (e.g., increased D2 receptor number, increased DA synthesis capacity, etc.) [38,39]. Thus, the simple model of abnormally high DA transmission could not explain the pathophysiology of schizophrenia or the therapeutic actions of antipsychotic medications.

The mechanism of action of antipsychotic drugs was advanced by the utilization of animal models to study schizophrenia [40]. The study of a disrupted circuit in animals approximates the dysregulated system present in patients with schizophrenia derived from clinical studies, such as the hippocampal dysfunction [41] that will be discussed here. This is essential because naïve animals present a high degree of homeostasis, which is not observed in animal models of disorders [42,43,44]. However, animal models are limited and can only approximate the pathological state in humans, but if based on clinical observations are essential to give insights into the mechanism of action of novel drug candidates [45]. Here, we focus on the neurodevelopmental model to study schizophrenia based on the administration of the DNA alkylating agent methyl azoxy methanol acetate (MAM) given to pregnant rodents at gestational day 17, and testing them as adults [42,46]. Gestational day 17 was chosen to approximate the human second trimester, which is a period of vulnerability in which infections or trauma will increase the risk of developing schizophrenia in the offspring [47]. We found that the adult offspring of MAM-treated dams could recapitulate a number of behavioral, pharmacological, neuroanatomical, and neural activity states that have been observed in schizophrenia patients [42,46]. This model was important to provide insights into the mechanism of action of antipsychotic drugs. For example, treatments with antipsychotic drugs are reported to be effective after a few days of administration in MAM rats [48], which is similar to that observed in patients with schizophrenia [49]. However, antipsychotics are only effective after 3–4 weeks of treatment in naïve rats [50,51].

## 2. Mechanism of Action of D2 Antagonist Antipsychotic Medications

A breakthrough came from animal studies of the actions of repeated antipsychotic drug administration on the DA system. In recordings from identified DA neurons in the brains of rats, it was found that 3+ weeks of treatment with first-generation antipsychotic drugs led to an inactivation of DA neuron firing—a phenomenon known as depolarization block [50,52,53]. It was found that the first-generation drugs caused depolarization block in both the nigrostriatal and mesolimbic DA system [50,52,54,55]. The nigrostriatal dopaminergic pathway projecting from substantia nigra compacta to the dorsal striatum is known to regulate motor control, and the mesolimbic pathway regulates projecting from the ventral tegmental area to the ventral striatum and limbic system, which are linked to reward, motivation, and emotion [56]. Interestingly, the second-generation drugs, which did not have extrapyramidal side effects but still exhibited therapeutic efficacy, only caused depolarization block in the mesolimbic psychosis-related DA system but not in the extrapyramidal nigrostriatal DA system [51,57,58]. Finally, it was shown that a compound with limited antipsychotic actions but prominent extrapyramidal actions, metoclopramide [59], only produced depolarization block in the nigrostriatal system but not the mesolimbic system [57]. Therefore, it was established that repeated antipsychotic drug-induced depolarization block of the mesolimbic system was associated with antipsychotic efficacy, and depolarization block of the nigrostriatal system with extrapyramidal side effects [54,57,58]. While this was a powerful correlation, there were some caveats: (1) antipsychotic drugs do not need to be administered for weeks to obtain therapeutic efficacy in patients, with onset being observed within 24 h of drug treatment [60]; (2) there was still no evidence for a hyperactive DA system; and (3) DA neuron depolarization block is not the normal state of the DA system.

One potential issue with these preclinical experiments is that they were performed on normal rats. It is well known that the brain has extensive homeostatic mechanisms that can compensate for the continued presence of a drug, and it was likely that the delayed action in the normal animals may have been due to these compensatory mechanisms [40]. What about animal models of schizophrenia, in which the system is already disrupted? We have proposed that in a disrupted system, in which homeostatic processes are either disrupted or ineffective, a therapeutic agent would have significantly different actions as compared to a normal system [40]. For this reason, it is essential to test these agents in an animal model that can approximate at least some of the circuit disruptions that clinical studies have found to also be altered in the schizophrenia brain [40,45].

**Figure 1:**
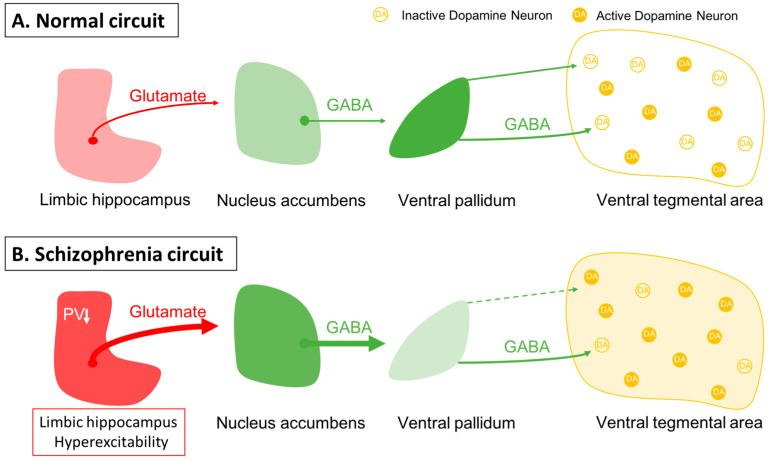
Dopamine neuron activity is driven by a pacemaker conductance that maintains their firing, which is offset by inhibition from the ventral pallidum. (**A**) In the baseline state, approximately half of the dopamine neurons are spontaneously firing, with the other half held in a hyperpolarized state due to GABAergic inhibition from the ventral pallidum. Activity in the limbic hippocampus provides an excitatory drive to the nucleus accumbens, which in turn can inhibit the ventral pallidum to modulate the inhibitory drive on ventral tegmental area dopamine neurons [78]. (**B**) In schizophrenia, a loss of parvalbumin GABAergic neurons in the limbic hippocampus causes this region to be tonically hyperactive; this leads to increased accumbens inhibition of the ventral pallidum. This releases the dopamine neurons from inhibition, causing the entire population of dopamine neurons to be in the active, responsive state.

**Figure 2:**
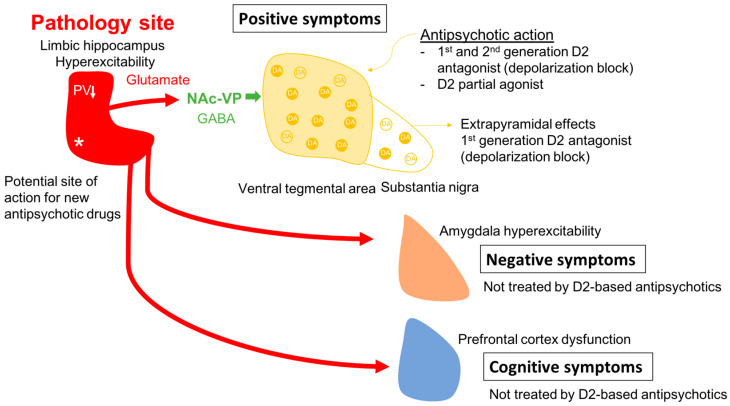
In schizophrenia, a loss of parvalbumin GABAergic neurons in the limbic hippocampus, via a circuit involving the nucleus accumbens and ventral pallidum leads to a disinhibition of VTA dopamine neurons and dopamine overdrive in the associative striatum; this appears to underlie the positive psychotic features of the disorder. By causing depolarization block of the ventral tegmental dopamine neurons, first- and second-generation antipsychotic drugs reverse dopamine neuron hyperactivity to relieve psychosis; in addition, first-generation drugs also cause depolarization block in the substantia nigra dopamine neurons to lead to extrapyramidal side effects. However, when the hippocampus is hyperactive and dysrhythmic, it can also lead to pathological activity changes in its other targets. Thus, it will impact the amygdala-cingulate cortex to lead to negative symptoms, and the prefrontal cortex to induce cognitive deficits; all characteristics of schizophrenia that are not effectively treated by dopamine antagonist first- and second-generation antipsychotic drugs. Novel mechanism antipsychotic drugs that act directly at the site of pathology in the hippocampus could be effective at reversing the negative and cognitive deficits as well as the positive symptoms of schizophrenia. * Site of pathology in schizophrenia; ↓ PV reduction in the hippocampus.

One issue in this approach is identifying an appropriate animal model. While it is clear that one cannot precisely replicate a complex uniquely human disorder like schizophrenia in a rodent, one can use clinical studies to guide development of an appropriate model and evaluate its functional validity. Evidence shows that schizophrenia likely has a strong genetic component, with the heritability of schizophrenia a function of number of shared genes in families [1,2]. In addition, there is clear evidence that environmental factors can also engender susceptibility to schizophrenia [1,3,4]. Furthermore, studies by Weinberger and others have shown that disruption of hippocampal function early in life will result in a rodent model that can recapitulate a number of features of schizophrenia [61,62,63,64,65,66]. In particular, imaging studies in schizophrenia patients have revealed hyperactivity in the limbic hippocampus [67], which is associated with an increase fluorodopa uptake in the associative striatum [68,69]. The associative striatum is the area of the striatum that receives inputs from associative areas of the neocortex and which correspond anatomically to the medial caudate-putamen segments of the striatum [70,71,72]. Similarly, in the MAM rats, we observed hyperactivity in the ventral limbic hippocampus and an increase in ventral tegmental area DA neuron activity [73]. This increase in DA neuron activity would thus be consistent with the increased fluorodopa uptake, since fluorodopa uptake is a metric of the number of active terminals [42,68], and we observed an increase in the number of DA neurons driving these terminals [42,46]. The hyperactivity of the hippocampus appears to be driven by a loss of parvalbumin-containing gamma-aminobutyric acidergic (GABAergic) interneurons, which is observed in postmortem schizophrenia brains [74,75] as well as in the MAM model of schizophrenia [76,77]. Furthermore, we showed that activation of the ventral hippocampus would, through a circuit involving the nucleus accumbens and the ventral pallidum, lead to an increase in the number of active DA neurons in the ventral tegmentum [78] (Figure 1). Thus, we propose that the overactive DA system involved in schizophrenia psychosis is due to loss of parvalbumin inhibition of the ventral hippocampus and consequent overdrive of the DA system. Importantly, while the overdriven DA system is likely the source of the psychotic positive symptoms of schizophrenia [79,80], the limbic hippocampus also exhibits projections to areas involved in affective regulation (e.g., the amygdala) and cognition (i.e., the prefrontal cortex) [81,82,83] (Figure 2). Thus, while targeting the DA system may help to relieve psychosis, it will not impact the negative and cognitive symptoms of this disorder.

**Figure 3:**
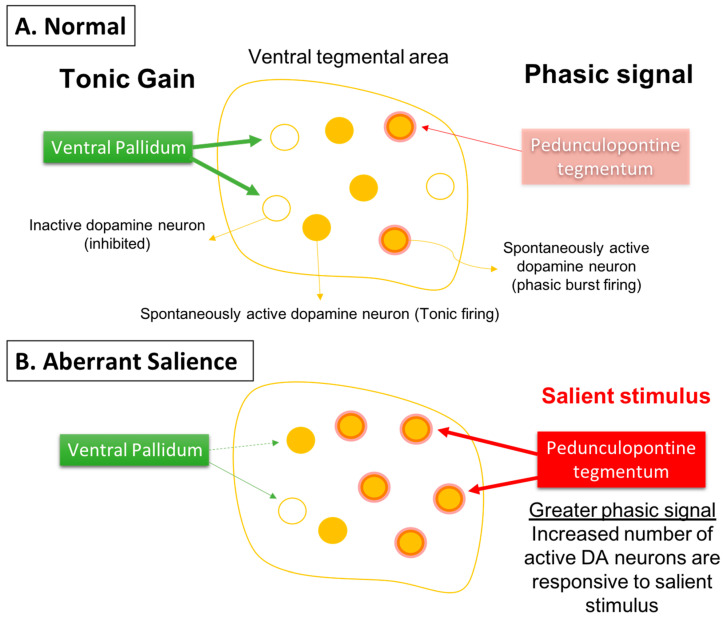
Phasic dopamine neuron burst firing is believed to be the behaviorally salient output of the dopamine system. (**A**) Burst firing is driven by a glutamate input from the pedunculopontine tegmentum acting on dopamine neuron n-methyl-D-aspartate (NMDA) receptors to drive burst firing. However, for NMDA to drive burst firing, the neuron must be in a depolarized, spontaneously active state; otherwise in hyperpolarized, inactive neurons there is a magnesium blockade of the NMDA channel. Therefore, only spontaneously active dopamine neurons can be driven by the pedunculopontine tegmentum into burst firing. The ventral pallidum, by controlling the number of dopamine neurons active, can determine the level of amplification, or the gain, of the phasic response. This is thought to be adjusted depending on the demands of the environment; in highly salient or dangerous conditions, the hippocampus increases the number of dopamine neurons in the responsive, active state, thereby enabling a salient stimulus to activate the pedunculopontine tegmental-driven burst firing across a large number of dopamine neurons, facilitating an immediate response to the threat. (**B**) In the case of schizophrenia, an overactive hippocampus removes tonic inhibitory drive of dopamine neurons, causing a massive increase in the number of responsive neurons independent of environmental contingencies. Under these conditions, both salient and nonsalient stimuli will cause a maximal phasic response. Therefore, with an overdriven dopamine system, every stimulus will be perceived as a threat, causing the patient to be overwhelmed and unable to filter salient from nonsalient stimuli. This leads to a state of aberrant salience, or the inappropriate attribution of salience to a normally benign object.

Given that the DA neurons are not hyperactive individually, but instead there are more of the neurons active, this would cause a stimulus that increases DA neuron firing and would have a substantially greater effect. Specifically, if the DA system is hyper-responsive to stimuli, this would cause an inappropriately high DA response to what may be benign stimuli, a condition known as aberrant salience [84,85,86]. This would lead to inappropriate attribution of threat or salience to benign stimuli (i.e., delusions) (Figure 3).

How would D2 antagonists help to alleviate this condition? In the normal animal, administration of antipsychotic drugs, by blocking postsynaptic D2 receptors, would cause a feedback activation of the DA system that, over weeks, results in DA neuron depolarization block. However, in the schizophrenia patient, the DA system is already in a hyperactive state [53]. In the MAM rats, we found that administration of a D2 antagonist, unlike in a normal animal, would add to the present hyper-responsive DA system, with the result that depolarization block develops very soon after drug administration [87]. This is consistent with the clinical literature, with the antipsychotic properties produced rapidly after antipsychotic medication initiation. Moreover, the more psychotic the patient, the more rapid the onset of therapeutic action [60,88]. Again, this is consistent with the MAM rat, in that the more overdriven the DA system is initially, the more rapidly will addition of a D2 antagonist produce depolarization block. By producing depolarization block and inactivation of DA neuron firing, this would alleviate the pathological increase in the number of DA neurons active and hyper-responsiveness to stimuli.

This provides a circuit-based assessment of the mechanism of antipsychotic drug treatment alleviation of psychosis [89]. However, it also exposes several caveats: (1) the “normal” state of the DA system is not depolarization block and this may be why antipsychotic drugs produce negative affective states, which may contribute to low patient compliance [90]; and (2) this will impact the DA system not by treating schizophrenia at the site of pathology (i.e., the limbic hippocampus), but instead 5 synapses downstream from the defect [86]. Thus, while the D2 blocking antipsychotic drug may alleviate psychosis, it would be ineffective in treating negative and cognitive deficits of this disorder.

How can some of these issues be circumvented? One more recent development is the use of partial DA agonist drugs, such as aripiprazole or brexpiprazole [91]. These drugs will occupy D2 receptors but, rather than producing a complete blockade, will produce a partial activation of the receptor [92]. Thus, these drugs will act by preventing overstimulation of D2 receptors while providing a baseline level of stimulation. For this reason, these drugs are typically administered at doses that occupy D2 receptors approximately 95% without producing excessive blockade-induced negative affect. We found that, unlike first- and second-generation antipsychotic drugs [53], the partial agonist aripiprazole appears to directly inhibit DA neuron activity without inducing depolarization block [93]. Therefore, while an improvement over receptor blockade-induced depolarization block, the drugs will nonetheless fail to alleviate the negative and cognitive deficits associated with schizophrenia, and will still impact the normal function of the DA system.

## 3. Novel Target Agents

As outlined above, current antipsychotic agents, while effective at treating psychosis, are not well-tolerated by patients and are not effective at alleviating negative and cognitive symptoms of the disorder, which likely underlies low patient compliance [94,95,96]. A more effective approach would be to target the site of pathology proposed to drive the schizophrenia state. As stated above, the current model suggests that parvalbumin neuron loss in the limbic hippocampus is driving the pathological state. Therefore, one potential mechanism to alleviate this dysfunction would be to either increase GABAergic inhibition at the parvalbumin-hippocampal pyramidal neuron site, or to directly decrease excitability of the hippocampal neurons. There are several compounds that were developed to address this. First, Lilly developed an mGluR2 3-agonist, pomaglumetad, a drug to target the hippocampal hyperexcitability [97]. Another compound was developed by Roche, a glycine uptake inhibitor [98] to increase glutamatergic NMDA drive on the parvalbumin interneurons. A third drug developed by Pfizer was a phosphodiesterase 10 inhibitor [99] to diminish the postsynaptic actions of the overactive glutamatergic system. In each case, the compounds showed significant efficacy in animal models of these disorders [98,100,101]. Indeed, in the MAM rats, pomaglumetad was found to be highly effective in normalizing hippocampal activity and DA neuron firing [100]. Furthermore, these compounds showed significant promise in the early phase trials [102,103,104,105]. However, in every case, the compounds failed to show separation from placebo in the multicenter clinical trials [106,107,108]. A conclusion drawn from these studies was that one cannot predict the clinical efficacy on the basis of animal models. However, the trial design and logic had a major flaw: whereas in the animal models, the first drug tested was the target compound, in the multicenter trials, these drugs were tested on long-term schizophrenia patients that had been withdrawn from the drug for only 1–2 weeks, which is the maximum that a therapeutic agent can be ethically withdrawn. The problem is, while this may be sufficient to wash out the compound from the system, it does not return the system to normal. Long-term D2 blockade will result in D2 supersensitivity; therefore, once the drug is withdrawn, a normally active DA system would still have a pathologically augmented postsynaptic response. Therefore, once a D2 antagonist is withdrawn and the supersensitive D2 receptors uncovered, the only drug that can act is another D2 antagonist [109].

We had shown that this is the case using another novel compound, a GABA A alpha 5-positive allosteric modulator. While GABA A synapses are present throughout the brain, the alpha 5 subunit is expressed primarily in the amygdala and the hippocampus, regions in which there is substantial parvalbumin neuron loss [76,77,110,111]. Administration of a novel GABA A alpha 5 positive allosteric modulator had no effect in normal rats; however, in MAM rats it rapidly normalized DA neuron activity, reversed amphetamine-induced hyperlocomotion, and restored hippocampal activity to baseline [112]. However, after administering the D2 antagonist antipsychotic drug haloperidol for only 3 weeks, which was sufficient to induce supersensitivity, this drug was ineffective at restoring amphetamine responses [87].

These data demonstrate that, in order to adequately evaluate a novel compound, one must test it on a system that is not already perturbed by prior drug administration. So how would one run a clinical trial under such conditions? There are 3 possibilities that may be evaluated: (1) One can test the drug on drug-naïve first episode patients. While this may be problematic with a novel, untested compound, Lilly’s analysis of clinical data on pomaglumetad revealed that the drug was most effective on patients in the early stages of the disorder [104], a time when supersensitivity may not have fully developed. (2) Another possibility is that, although we cannot ethically withdraw patients from an antipsychotic drug for more than 2 weeks, in actuality the patients withdraw themselves all the time, given that there is a nearly 70% noncompliance with their medication [113,114,115,116]. Therefore, targeting patient populations that have demonstrated long-term noncompliance may be an effective strategy. (3) Finally, one could use patients that were on medications that do not induce supersensitivity. Data suggest that this may be the partial agonists, given that these drugs stimulate D2 receptors at least partially [91] and also do not induce DA neuron depolarization block [93].

On this basis, to identify effective novel compounds, it would be necessary to alter the manner in which clinical trials are performed. While the prior model may have been effective in identifying DA antagonist medications, they do not appear to be effective in evaluating novel and potentially more effective treatments.

Another approach would be to limit the overactivity of hippocampal pyramidal neurons. One compound that can achieve this effect is evenamide, which will normalize excess glutamate release without affecting baseline levels, which would be ideal for normalizing overactive hippocampal neurons [117]. This drug was found to be effective in reversing selective symptoms produced by amphetamines, phencyclidine, MK-801, or ketamine [118,119], all of which act via hippocampal hyperactivity [73,120,121]. When tested as an adjunct to standard-of-care treatment of patients that were showing worsening on their current medications, evenamide produced a significant improvement in positive and negative symptom scales [117]. Testing whether this will also be effective as a monotherapy could yield important insights into how normalizing glutamate overdrive may reverse schizophrenia symptomatology.

## 4. Conclusions

Based on preclinical data, to identify effective novel compounds, it would be necessary to alter the manner in which clinical trials are performed. While the prior model may have been effective in identifying DA antagonist medications, they do not appear to be effective in evaluating novel and potentially more effective treatments. Current trial designs may be effective at identifying additional D2 antagonist drugs, but not compounds that have a unique site of action. By targeting the site of functional deficits related to hippocampal overdrive, one may be in a position to provide more effective treatments that are better tolerated and can address the broad range of schizophrenia symptomatology.

## Data Availability

No new data were created or analyzed in this study. Data sharing is not applicable to this article.

## References

[1] Miller P., Lawrie S.M., Hodges A., Clafferty R., Cosway R., Johnstone E.C. (2001). Genetic liability, illicit drug use, life stress and psychotic symptoms: Preliminary findings from the Edinburgh study of people at high risk for schizophrenia. Soc. Psychiatry Psychiatr. Epidemiol..

[2] Henriksen M.G., Nordgaard J., Jansson L.B. (2017). Genetics of Schizophrenia: Overview of Methods, Findings and Limitations. Front. Hum. Neurosci..

[3] Popovic D., Schmitt A., Kaurani L., Senner F., Papiol S., Malchow B., Fischer A., Schulze T.G., Koutsouleris N., Falkai P. (2019). Childhood Trauma in Schizophrenia: Current Findings and Research Perspectives. Front. Neurosci..

[4] Berthelot N., Garon-Bissonnette J., Jomphe V., Doucet-Beaupré H., Bureau A., Maziade M. (2022). Childhood trauma may increase risk of psychosis and mood disorder in genetically high-risk children and adolescents by enhancing the accumulation of risk indicators. Schizophr. Bull. Open.

[5] McCutcheon R.A., Reis Marques T., Howes O.D. (2020). Schizophrenia—An Overview. JAMA Psychiatry.

[6] Messias E.L., Chen C.-Y., Eaton W.W. (2007). Epidemiology of schizophrenia: Review of findings and myths. Psychiatr. Clin. N. Am..

[7] Charlson F.J., Ferrari A.J., Santomauro D.F., Diminic S., Stockings E., Scott J.G., McGrath J.J., Whiteford H.A. (2018). Global Epidemiology and Burden of Schizophrenia: Findings from the Global Burden of Disease Study 2016. Schizophr. Bull..

[8] Kadakia A., Catillon M., Fan Q., Williams G.R., Marden J.R., Anderson A., Kirson N., Dembek C. (2022). The Economic Burden of Schizophrenia in the United States. J. Clin. Psychiatry.

[9] Gomes F.V., Rincón-Cortés M., Grace A.A. (2016). Adolescence as a period of vulnerability and intervention in schizophrenia: Insights from the MAM model. Neurosci. Biobehav. Rev..

[10] Ban T.A. (2007). Fifty years chlorpromazine: A historical perspective. Neuropsychiatr. Dis. Treat..

[11] Shen W.W. (1999). A history of antipsychotic drug development. Compr. Psychiatry.

[12] Laborit H., Huguenard P. (1951). Artificial hibernation by pharmacodynamic and physical means, in surgery. J. Chir..

[13] Casey J.F., Bennett I.F., Lindley C.J., Hollister L.E., Gordon M.H., Springer N.N. (1960). Drug therapy in schizophrenia. A controlled study of the relative effectiveness of chlorpromazine, promazine, phenobarbital, and placebo. AMA Arch. Gen. Psychiatry.

[14] Elkes J., Elkes C. (1954). Effect of chlorpromazine on the behavior of chronically overactive psychotic patients. Br. Med. J..

[15] Lehmann H.E., Hanrahan G.E. (1954). Chlorpromazine; new inhibiting agent for psychomotor excitement and manic states. AMA Arch. Neurol. Psychiatry.

[16] Delay J., Deniker P., Harl J.M. (1952). Therapeutic method derived from hiberno-therapy in excitation and agitation states. Ann. Med. Psychol..

[17] Hamon, Paraire, Velluz (1952). Effect of R. P. 4560 on maniacal agitation. Ann. Med. Psychol..

[18] Creese I., Burt D.R., Snyder S.H. (1976). Dopamine receptor binding predicts clinical and pharmacological potencies of antischizophrenic drugs. Science.

[19] Seeman P., Lee T., Chau-Wong M., Wong K. (1976). Antipsychotic drug doses and neuroleptic/dopamine receptors. Nature.

[20] Seeman P. (1992). Dopamine receptor sequences. Therapeutic levels of neuroleptics occupy D2 receptors, clozapine occupies D4. Neuropsychopharmacology.

[21] Abi-Dargham A., Van De Giessen E., Slifstein M., Kegeles L.S., Laruelle M. (2009). Baseline and Amphetamine-Stimulated Dopamine Activity Are Related in Drug-Naïve Schizophrenic Subjects. Biol. Psychiatry.

[22] Laruelle M., Abi-Dargham A., Van Dyck C.H., Gil R., D’Souza C.D., Erdos J., McCance E., Rosenblatt W., Fingado C., Zoghbi S.S. (1996). Single photon emission computerized tomography imaging of amphetamine-induced dopamine release in drug-free schizophrenic subjects. Proc. Natl. Acad. Sci. USA.

[23] Laruelle M., Abi-Dargham A., Gil R., Kegeles L., Innis R. (1999). Increased dopamine transmission in schizophrenia: Relationship to illness phases. Biol. Psychiatry.

[24] Gründer G., Cumming P. (2016). The Dopamine Hypothesis of Schizophrenia. The Neurobiology of Schizophrenia.

[25] Brisch R., Saniotis A., Wolf R., Bielau H., Bernstein H.-G., Steiner J., Bogerts B., Braun K., Jankowski Z., Kumaratilake J. (2014). The role of dopamine in schizophrenia from a neurobiological and evolutionary perspective: Old fashioned, but still in vogue. Front. Psychiatry.

[26] Haase H.J., Janssen P.A.J. (1958). The Action of Neuroleptic Drugs: A Psychiatric, Neurologic and Pharmacological Investigation.

[27] Hippius H. (1989). The history of clozapine. Psychopharmacology.

[28] Crilly J. (2007). The history of clozapine and its emergence in the US market: A review and analysis. Hist. Psychiatry.

[29] Kane J., Honigfeld G., Singer J., Meltzer H. (1988). Clozapine for the treatment-resistant schizophrenic. A double-blind comparison with chlorpromazine. Arch. Gen. Psychiatry.

[30] Meltzer H.Y., Matsubara S., Lee J.C. (1989). Classification of typical and atypical antipsychotic drugs on the basis of dopamine D-1, D-2 and serotonin2 pKi values. J. Pharmacol. Exp. Ther..

[31] Terry A.V. (2008). Role of the central cholinergic system in the therapeutics of schizophrenia. Curr. Neuropharmacol..

[32] Farde L., Nordström A.L., Wiesel F.A., Pauli S., Halldin C., Sedvall G. (1992). Positron emission tomographic analysis of central D1 and D2 dopamine receptor occupancy in patients treated with classical neuroleptics and clozapine. Relation to extrapyramidal side effects. Arch. Gen. Psychiatry.

[33] Kapur S., Remington G., Jones C., Wilson A., DaSilva J., Houle S., Zipursky R. (1996). High levels of dopamine D2 receptor occupancy with low-dose haloperidol treatment: A PET study. Am. J. Psychiatry.

[34] Nordström A.L., Farde L., Wiesel F.A., Forslund K., Pauli S., Halldin C., Uppfeldt G. (1993). Central D2-dopamine receptor occupancy in relation to antipsychotic drug effects: A double-blind PET study of schizophrenic patients. Biol. Psychiatry.

[35] Kapur S., Zipursky R., Jones C., Remington G., Houle S. (2000). Relationship between dopamine D(2) occupancy, clinical response, and side effects: A double-blind PET study of first-episode schizophrenia. Am. J. Psychiatry.

[36] Joel D., Weiner I., Feldon J. (1997). Electrolytic lesions of the medial prefrontal cortex in rats disrupt performance on an analog of the Wisconsin Card Sorting Test, but do not disrupt latent inhibition: Implications for animal models of schizophrenia. Behav. Brain Res..

[37] Kokkinidis L., Anisman H. (1981). Amphetamine psychosis and schizophrenia: A dual model. Neurosci. Biobehav. Rev..

[38] Correll C.U., Rubio J.M., Kane J.M. (2018). What is the risk-benefit ratio of long-term antipsychotic treatment in people with schizophrenia?. World Psychiatry.

[39] Miyamoto S., Duncan G.E., Marx C.E., Lieberman J.A. (2005). Treatments for schizophrenia: A critical review of pharmacology and mechanisms of action of antipsychotic drugs. Mol. Psychiatry.

[40] Uliana D.L., Gomes F.V., Grace A.A. (2023). Update on current animal models for schizophrenia: Are they still useful?. Curr. Opin. Psychiatry.

[41] Wegrzyn D., Juckel G., Faissner A. (2022). Structural and Functional Deviations of the Hippocampus in Schizophrenia and Schizophrenia Animal Models. Int. J. Mol. Sci..

[42] Modinos G., Allen P., Grace A.A., McGuire P. (2015). Translating the MAM model of psychosis to humans. Trends Neurosci..

[43] Meyer U. (2014). Prenatal poly(i:C) exposure and other developmental immune activation models in rodent systems. Biol. Psychiatry.

[44] Gomes F.V., Zhu X., Grace A.A. (2019). Stress during critical periods of development and risk for schizophrenia. Schizophr. Res..

[45] Uliana D.L., Zhu X., Gomes F.V., Grace A.A. (2022). Using animal models for the studies of schizophrenia and depression: The value of translational models for treatment and prevention. Front. Behav. Neurosci..

[46] Moore H., Jentsch J.D., Ghajarnia M., Geyer M.A., Grace A.A. (2006). A neurobehavioral systems analysis of adult rats exposed to methylazoxymethanol acetate on E17: Implications for the neuropathology of schizophrenia. Biol. Psychiatry.

[47] Barr C.E., Mednick S.A., Munk-Jorgensen P. (1990). Exposure to influenza epidemics during gestation and adult schizophrenia. A 40-year study. Arch. Gen. Psychiatry.

[48] Valenti O., Cifelli P., Gill K.M., Grace A.A. (2011). Antipsychotic drugs rapidly induce dopamine neuron depolarization block in a developmental rat model of schizophrenia. J. Neurosci..

[49] Agid O., Seeman P., Kapur S. (2006). The “delayed onset” of antipsychotic action--an idea whose time has come and gone. J. Psychiatry Neurosci..

[50] Bunney B.S., Grace A.A. (1978). Acute and chronic haloperidol treatment: Comparison of effects on nigral dopaminergic cell activity. Life Sci..

[51] Chiodo L.A., Bunney B.S. (1983). Typical and atypical neuroleptics: Differential effects of chronic administration on the activity of A9 and A10 midbrain dopaminergic neurons. J. Neurosci..

[52] Grace A.A., Bunney B.S. (1986). Induction of depolarization block in midbrain dopamine neurons by repeated administration of haloperidol: Analysis using in vivo intracellular recording. J. Pharmacol. Exp. Ther..

[53] Grace A.A., Bunney B.S., Moore H., Todd C.L. (1997). Dopamine-cell depolarization block as a model for the therapeutic actions of antipsychotic drugs. Trends Neurosci..

[54] Grace A.A. (1992). The depolarization block hypothesis of neuroleptic action: Implications for the etiology and treatment of schizophrenia. J. Neural. Transm. Suppl..

[55] Lane R.F., Blaha C.D. (1987). Chronic haloperidol decreases dopamine release in striatum and nucleus accumbens in vivo: Depolarization block as a possible mechanism of action. Brain Res. Bull.

[56] Luo S.X., Huang E.J. (2016). Dopaminergic Neurons and Brain Reward Pathways: From Neurogenesis to Circuit Assembly. Am. J. Pathol..

[57] White F.J., Wang R.Y. (1983). Differential effects of classical and atypical antipsychotic drugs on A9 and A10 dopamine neurons. Science.

[58] Goldstein J.M., Litwin L.C., Sutton E.B., Malick J.B. (1993). Seroquel: Electrophysiological profile of a potential atypical antipsychotic. Psychopharmacology.

[59] Harrington R.A., Hamilton C.W., Brogden R.N., Linkewich J.A., Romankiewicz J.A., Heel R.C. (1983). Metoclopramide. An updated review of its pharmacological properties and clinical use. Drugs.

[60] Kapur S., Arenovich T., Agid O., Zipursky R., Lindborg S., Jones B. (2005). Evidence for onset of antipsychotic effects within the first 24 hours of treatment. Am. J. Psychiatry.

[61] Weinberger D.R. (1987). Implications of normal brain development for the pathogenesis of schizophrenia. Arch. Gen. Psychiatry.

[62] Lipska B.K., Jaskiw G.E., Weinberger D.R. (1993). Postpubertal emergence of hyperresponsiveness to stress and to amphetamine after neonatal excitotoxic hippocampal damage: A potential animal model of schizophrenia. Neuropsychopharmacology.

[63] Chambers R.A., Moore J., McEvoy J.P., Levin E.D. (1996). Cognitive effects of neonatal hippocampal lesions in a rat model of schizophrenia. Neuropsychopharmacology.

[64] Becker A., Grecksch G., Bernstein H.G., Höllt V., Bogerts B. (1999). Social behaviour in rats lesioned with ibotenic acid in the hippocampus: Quantitative and qualitative analysis. Psychopharmacology.

[65] Grecksch G., Bernstein H.G., Becker A., Höllt V., Bogerts B. (1999). Disruption of latent inhibition in rats with postnatal hippocampal lesions. Neuropsychopharmacology.

[66] Tseng K.Y., Chambers R.A., Lipska B.K. (2009). The neonatal ventral hippocampal lesion as a heuristic neurodevelopmental model of schizophrenia. Behav. Brain Res..

[67] Heckers S., Konradi C. (2010). Hippocampal pathology in schizophrenia. Curr. Top Behav. Neurosci..

[68] Howes O.D., Kambeitz J., Kim E., Stahl D., Slifstein M., Abi-Dargham A., Kapur S. (2012). The Nature of Dopamine Dysfunction in Schizophrenia and What This Means for Treatment: Meta-analysis of Imaging Studies. Arch. Gen. Psychiatry.

[69] McGowan S., Lawrence A.D., Sales T., Quested D., Grasby P. (2004). Presynaptic Dopaminergic Dysfunction in Schizophrenia: A Positron Emission Tomographic [^18^F]Fluorodopa Study. Arch. Gen. Psychiatry.

[70] Joel D., Weiner I. (1994). The organization of the basal ganglia-thalamocortical circuits: Open interconnected rather than closed segregated. Neuroscience.

[71] Parent A., Hazrati L.-N. (1995). Functional anatomy of the basal ganglia. I. The cortico-basal ganglia-thalamo-cortical loop. Brain Res. Rev..

[72] Joel D., Weiner I. (2000). The connections of the dopaminergic system with the striatum in rats and primates: An analysis with respect to the functional and compartmental organization of the striatum. Neuroscience.

[73] Lodge D.J., Grace A.A. (2007). Aberrant hippocampal activity underlies the dopamine dysregulation in an animal model of schizophrenia. J. Neurosci..

[74] Zhang Z.J., Reynolds G.P. (2002). A selective decrease in the relative density of parvalbumin-immunoreactive neurons in the hippocampus in schizophrenia. Schizophr. Res..

[75] Konradi C., Yang C.K., Zimmerman E.I., Lohmann K.M., Gresch P., Pantazopoulos H., Berretta S., Heckers S. (2011). Hippocampal interneurons are abnormal in schizophrenia. Schizophr. Res..

[76] Lodge D.J., Behrens M.M., Grace A.A. (2009). A loss of parvalbumin-containing interneurons is associated with diminished oscillatory activity in an animal model of schizophrenia. J. Neurosci..

[77] Gill K.M., Grace A.A. (2014). Corresponding decrease in neuronal markers signals progressive parvalbumin neuron loss in MAM schizophrenia model. Int. J. Neuropsychopharmacol..

[78] Floresco S.B., Todd C.L., Grace A.A. (2001). Glutamatergic afferents from the hippocampus to the nucleus accumbens regulate activity of ventral tegmental area dopamine neurons. J. Neurosci..

[79] Wong D.F., Wagner H.N., Tune L.E., Dannals R.F., Pearlson G.D., Links J.M., Tamminga C.A., Broussolle E.P., Ravert H.T., Wilson A.A. (1986). Positron emission tomography reveals elevated D2 dopamine receptors in drug-naive schizophrenics. Science.

[80] Tost H., Alam T., Meyer-Lindenberg A. (2010). Dopamine and psychosis: Theory, pathomechanisms and intermediate phenotypes. Neurosci. Biobehav. Rev..

[81] Ghoshal A., Conn P.J. (2015). The hippocampo-prefrontal pathway: A possible therapeutic target for negative and cognitive symptoms of schizophrenia. Future Neurol..

[82] O’Donnell P., Grace A.A. (1995). Synaptic interactions among excitatory afferents to nucleus accumbens neurons: Hippocampal gating of prefrontal cortical input. J. Neurosci..

[83] O’Donnell P., Lewis B.L., Weinberger D.R., Lipska B.K. (2002). Neonatal hippocampal damage alters electrophysiological properties of prefrontal cortical neurons in adult rats. Cereb. Cortex.

[84] Kapur S. (2003). Psychosis as a state of aberrant salience: A framework linking biology, phenomenology, and pharmacology in schizophrenia. Am. J. Psychiatry.

[85] Sonnenschein S.F., Gomes F.V., Grace A.A. (2020). Dysregulation of Midbrain Dopamine System and the Pathophysiology of Schizophrenia. Front. Psychiatry.

[86] Grace A.A. (2016). Dysregulation of the dopamine system in the pathophysiology of schizophrenia and depression. Nat. Rev. Neurosci..

[87] Gill K.M., Cook J.M., Poe M.M., Grace A.A. (2014). Prior antipsychotic drug treatment prevents response to novel antipsychotic agent in the methylazoxymethanol acetate model of schizophrenia. Schizophr. Bull..

[88] Haddad P.M., Correll C.U. (2018). The acute efficacy of antipsychotics in schizophrenia: A review of recent meta-analyses. Ther Adv Psychopharmacol..

[89] Sonnenschein S.F., Grace A.A. (2020). Insights on current and novel antipsychotic mechanisms from the MAM model of schizophrenia. Neuropharmacology.

[90] Haddad P.M., Brain C., Scott J. (2014). Nonadherence with antipsychotic medication in schizophrenia: Challenges and management strategies. Patient Relat. Outcome Meas..

[91] Mohr P., Masopust J., Kopeček M. (2022). Dopamine Receptor Partial Agonists: Do They Differ in Their Clinical Efficacy?. Front. Psychiatry.

[92] Burris K.D., Molski T.F., Xu C., Ryan E., Tottori K., Kikuchi T., Yocca F.D., Molinoff P.B. (2002). Aripiprazole, a novel antipsychotic, is a high-affinity partial agonist at human dopamine D2 receptors. J. Pharmacol. Exp. Ther..

[93] Sonnenschein S.F., Gill K.M., Grace A.A. (2019). State-dependent effects of the D2 partial agonist aripiprazole on dopamine neuron activity in the MAM neurodevelopmental model of schizophrenia. Neuropsychopharmacology.

[94] Leucht S., Pitschel-Walz G., Abraham D., Kissling W. (1999). Efficacy and extrapyramidal side-effects of the new antipsychotics olanzapine, quetiapine, risperidone, and sertindole compared to conventional antipsychotics and placebo. A meta-analysis of randomized controlled trials. Schizophr. Res..

[95] Keefe R.S.E., Sweeney J.A., Gu H., Hamer R.M., Perkins D.O., McEvoy J.P., Lieberman J.A. (2007). Effects of olanzapine, quetiapine, and risperidone on neurocognitive function in early psychosis: A randomized, double-blind 52-week comparison. Am. J. Psychiatry.

[96] Keefe R.S.E., Bilder R.M., Davis S.M., Harvey P.D., Palmer B.W., Gold J.M., Meltzer H.Y., Green M.F., Capuano G., Stroup T.S. (2007). Neurocognitive effects of antipsychotic medications in patients with chronic schizophrenia in the CATIE Trial. Arch. Gen. Psychiatry.

[97] Rorick-Kehn L.M., Johnson B.G., Knitowski K.M., Salhoff C.R., Witkin J.M., Perry K.W., Griffey K.I., Tizzano J.P., Monn J.A., McKinzie D.L. (2007). In vivo pharmacological characterization of the structurally novel, potent, selective mGlu2/3 receptor agonist LY404039 in animal models of psychiatric disorders. Psychopharmacology.

[98] Alberati D., Moreau J.-L., Lengyel J., Hauser N., Mory R., Borroni E., Pinard E., Knoflach F., Schlotterbeck G., Hainzl D. (2012). Glycine reuptake inhibitor RG1678: A pharmacologic characterization of an investigational agent for the treatment of schizophrenia. Neuropharmacology.

[99] Menniti F.S., Chappie T.A., Schmidt C.J. (2020). PDE10A Inhibitors-Clinical Failure or Window Into Antipsychotic Drug Action?. Front. Neurosci..

[100] Sonnenschein S.F., Grace A.A. (2020). The mGluR2/3 agonist pomaglumetad methionil normalizes aberrant dopamine neuron activity via action in the ventral hippocampus. Neuropsychopharmacology.

[101] Jankowska A., Świerczek A., Wyska E., Gawalska A., Bucki A., Pawłowski M., Chłoń-Rzepa G. (2019). Advances in Discovery of PDE10A Inhibitors for CNS-Related Disorders. Part 1: Overview of the Chemical and Biological Research. Curr. Drug Targets.

[102] Umbricht D., Alberati D., Martin-Facklam M., Borroni E., Youssef E.A., Ostland M., Wallace T.L., Knoflach F., Dorflinger E., Wettstein J.G. (2014). Effect of bitopertin, a glycine reuptake inhibitor, on negative symptoms of schizophrenia: A randomized, double-blind, proof-of-concept study. JAMA Psychiatry.

[103] Macek T.A., McCue M., Dong X., Hanson E., Goldsmith P., Affinito J., Mahableshwarkar A.R. (2019). A phase 2, randomized, placebo-controlled study of the efficacy and safety of TAK-063 in subjects with an acute exacerbation of schizophrenia. Schizophr. Res..

[104] Kinon B.J., Millen B.A., Zhang L., McKinzie D.L. (2015). Exploratory analysis for a targeted patient population responsive to the metabotropic glutamate 2/3 receptor agonist pomaglumetad methionil in schizophrenia. Biol. Psychiatry.

[105] Adams D.H., Kinon B.J., Baygani S., Millen B.A., Velona I., Kollack-Walker S., Walling D.P. (2013). A long-term, phase 2, multicenter, randomized, open-label, comparative safety study of pomaglumetad methionil (LY2140023 monohydrate) versus atypical antipsychotic standard of care in patients with schizophrenia. BMC Psychiatry.

[106] Bugarski-Kirola D., Iwata N., Sameljak S., Reid C., Blaettler T., Millar L., Marques T.R., Garibaldi G., Kapur S. (2016). Efficacy and safety of adjunctive bitopertin versus placebo in patients with suboptimally controlled symptoms of schizophrenia treated with antipsychotics: Results from three phase 3, randomised, double-blind, parallel-group, placebo-controlled, multicentre studies in the SearchLyte clinical trial programme. Lancet Psychiatry.

[107] Walling D.P., Banerjee A., Dawra V., Boyer S., Schmidt C.J., DeMartinis N. (2019). Phosphodiesterase 10A Inhibitor Monotherapy Is Not an Effective Treatment of Acute Schizophrenia. J. Clin. Psychopharmacol..

[108] Adams D.H., Zhang L., Millen B.A., Kinon B.J., Gomez J.-C. (2014). Pomaglumetad Methionil (LY2140023 Monohydrate) and Aripiprazole in Patients with Schizophrenia: A Phase 3, Multicenter, Double-Blind Comparison. Schizophr. Res. Treatment.

[109] Servonnet A., Samaha A.-N. (2020). Antipsychotic-evoked dopamine supersensitivity. Neuropharmacology.

[110] Du Y., Grace A.A. (2016). Amygdala Hyperactivity in MAM Model of Schizophrenia is Normalized by Peripubertal Diazepam Administration. Neuropsychopharmacology.

[111] Zhu X., Grace A.A. (2022). Sex- and exposure age-dependent effects of adolescent stress on ventral tegmental area dopamine system and its afferent regulators. Mol. Psychiatry.

[112] Gill K.M., Lodge D.J., Cook J.M., Aras S., Grace A.A. (2011). A novel α5GABA(A)R-positive allosteric modulator reverses hyperactivation of the dopamine system in the MAM model of schizophrenia. Neuropsychopharmacology.

[113] Velligan D.I., Weiden P.J., Sajatovic M., Scott J., Carpenter D., Ross R., Docherty J.P. (2009). Expert Consensus Panel on Adherence Problems in Serious and Persistent Mental Illness The expert consensus guideline series: Adherence problems in patients with serious and persistent mental illness. J. Clin. Psychiatry.

[114] Lacro J.P., Dunn L.B., Dolder C.R., Leckband S.G., Jeste D.V. (2002). Prevalence of and risk factors for medication nonadherence in patients with schizophrenia: A comprehensive review of recent literature. J. Clin. Psychiatry.

[115] Cramer J.A., Rosenheck R. (1998). Compliance with medication regimens for mental and physical disorders. Psychiatr. Serv..

[116] Lieslehto J., Tiihonen J., Lähteenvuo M., Tanskanen A., Taipale H. (2022). Primary Nonadherence to Antipsychotic Treatment Among Persons with Schizophrenia. Schizophr. Bull..

[117] Anand R., Turolla A., Chinellato G., Roy A., Hartman R.D. (2023). Phase 2 results indicate evenamide, a selective modulator of glutamate release, is associated with remarkable clinically important long-term efficacy when added to an antipsychotic in patients with treatment-resistant schizophrenia (TRS). Int. J. Neuropsychopharmacol..

[118] Bortolato M., Faravelli L., Anand R. (2018). T36. The antipsychotic-like properties of evenamide (NW-3509) reflect the modulation of glutamatergic dysregulation. Schizophr. Bull..

[119] Faravelli R., Anand R., Forrest E. (2016). Evenamide (formerly NW-3509) targets new mechanisms, and represents a new approach to the management of untreated symptoms in schizophrenia. Eur. Neuropsychopharmacol..

[120] Lodge D.J., Grace A.A. (2008). Amphetamine activation of hippocampal drive of mesolimbic dopamine neurons: A mechanism of behavioral sensitization. J. Neurosci..

[121] White I.M., Whitaker C., White W. (2006). Amphetamine-induced hyperlocomotion in rats: Hippocampal modulation of the nucleus accumbens. Hippocampus.

